# Herbal oral care products for the prevention of ventilator-associated pneumonia: A systematic review and network meta-analysis of randomised trials

**DOI:** 10.1371/journal.pone.0304583

**Published:** 2024-06-07

**Authors:** SuWen Li, YanNan Huang, HongYin Xie

**Affiliations:** Faculty of Nursing, Hospital of Gannan Medical University, Ganzhou, Jiangxi province, China; Alexandria University Faculty of Nursing, EGYPT

## Abstract

**Background:**

The recommendation for Chlorhexidine (CHX) as a traditional oral care solution is decreasing, and herbal oral care products are being considered as a potential alternative. This network meta-analysis aims to determine if herbal oral care products for oral care in mechanically ventilated patients are superior to CHX and provide direction for future research by comparing the effectiveness of herbal oral care products currently available.

**Materials and methods:**

We searched for English-language published and grey literature sources of randomized clinical trials involving herbal oral care solutions in intensive care unit (ICU) oral care (until September 2023). The primary outcome was the incidence of ventilator-associated pneumonia (VAP); the secondary outcome was the oral microbiota quantity. Data were pooled by pairwise meta-analysis and Bayesian network meta-analysis. The risk of bias was assessed using the Cochrane risk of bias tool, and the certainty of evidence was evaluated using the GRADE framework.

**Results:**

Our network meta-analysis included 29 studies, and the results showed that Chinese herb (OR: 0.39, 95% CI: 0.2–0.75) and Miswak (OR: 0.27, 95% CI: 0.07–0.91) were more effective in reducing VAP incidence than CHX. In terms of reducing bacterial counts, Chinese herb (OR: 0.3, 95% CI: 0.19–0.48) was superior to CHX, and all herbal oral care products, including Persica^®^ (alcoholic extract of S. persica, Achillea millefolium, and Mentha spicata), Matrica^®^ (Chamomile extract), and Listerine^®^ (main components include Menthol, Thymol, and Eucalyptol), were better than saline in all aspects but without significant differences.

**Conclusion:**

Based on our network meta-analysis, we have observed that Chinese herbal medicine and Miswak are superior to CHX in reducing the incidence of VAP. However, the safety and feasibility of traditional Chinese herbal medicine require further high-quality research for validation. Simultaneously, Matrica^®^ demonstrates a significant reduction in microbial counts but does not exhibit a significant advantage in lowering the incidence of VAP. This observation aligns with the results of clinical double-blind trials. Therefore, we identify Miswak and Matrica^®^ as promising herbal oral care products with the potential to replace CHX. It is essential to emphasize that our study provides guidance for future research rather than conclusive determinations.

**Registration:**

PROSPERO no. CRD42023398022.

## Introduction

In recent years, with the development of clinical intensive care medicine, mechanical ventilation (MV) orotracheal intubation has been widely used in intensive care unit (ICU) [1]. However, the oral microbiota of MV patients begins to change on the first day of admission to the ICU, and pathogenic microorganisms quickly accumulate in the oral cavity, eventually leading to ventilator-associated pneumonia (VAP) [2]. VAP is defined as a pneumonia that occurs after 48 to 72 hours or later of endotracheal intubation. The diagnosis of this disease requires the fulfillment of ventilator-associated conditions (VAC) or infection-related ventilator-associated complications (IVAC) criteria, along with positive respiratory cultures or histopathological evidence [3]. It has been reported that 5%-40% of patients receiving invasive mechanical ventilation for more than 2 days develop VAP [4]. VAP remains an important disease in the ICU with significant economic burden, not only increasing mortality rates but also resulting in substantial costs. A recent cost evaluation from the Spain that the attributable cost of VAP was €20,965 [5]. Patients receiving mechanical ventilation are prone to rapid colonization of lower respiratory tract pathogens after intubation. Numerous studies over the past 20 years have shown that bacterial colonization of the oral-pharyngeal region is a key factor leading to respiratory infections in mechanically ventilated patients [6–9]. During intubation, colonized bacteria in the oral cavity and throat can migrate directly to the respiratory tract via the endotracheal tube, leading to VAP. Changes in saliva composition and function, altered oral pH, and increased plaque also occur in mechanically ventilated patients after intubation, which increases colonization of pathogenic bacteria in the oral-pharyngeal region and becomes the main source of pathogenic bacteria related to VAP [10].

Reduction of microbial counts in the oral cavity improves lung migration and colonization. As a common medicinal product, chlorhexidine (CHX) mouthwash is very effective in reducing pathogenic microorganisms including Streptococcus mutans and plaque [11]. However, with further research on CHX, many meta-analyses have found that CHX is only effective for patients undergoing major cardiac and vascular surgery [11, 12]. Moreover, CHX has side effects such as tooth staining, unpleasant taste, dry mouth, allergies, and burning sensation [13]. Recent reports have suggested that CHX may have potential adverse effects on oral mucosa and decrease bacterial sensitivity. There is also a potential association between CHX oral care and increased mortality [14]. A recent study conducted a hospital-wide retrospective observational cohort analysis of the impact of CHX oral care on hospital mortality and confirmed this association [15].

Currently, the "US guidelines for the prevention of VAP" have downgraded CHX oral care from a routine recommendation in all hospitals to only being recommended for hospitals that have implemented more basic prevention measures but still have a high incidence of VAP [16]. Doctors and critically ill patients in the ICU tend to look for other mouthwashes with beneficial effects similar to CHX while reducing adverse effects. Herbal mouthwash has been considered a suitable alternative to CHX due to its lower side effects of its extracts and ability to reduce bacterial count [17, 18].

To assess the effectiveness of herbal extracts and natural oral care products in lowering the incidence of VAP among ventilated patients, we performed a network meta-analysis (NMA) of randomized clinical trials. NMA is a statistical method that allows for the integration of direct and indirect comparisons of multiple treatment modalities within a single analysis, enabling estimation and ranking of their relative effectiveness. Our aim was to compare the efficacy of currently available herbal oral care products and provide guidance for future research and safer, more effective oral care protocols for ICU patients.

## Methods

The NMA has been prospectively registered with the International Prospective Register of Systematic Reviews (CRD42023398022). In addition, this study followed the Preferred Reporting Items for Systematic Reviews and Meta-Analyses Network Meta-Analysis (PRISMA-NMA) guidelines [19], and we adhered to the recommendations of the Cochrane Handbook for Systematic Reviews of Interventions.

### Search strategy

Two investigators independently searched relevant studies in this field from online databases, including China National Knowledge Infrastructure (CNKI), Wanfang Database, China Biology Medicine (CBM), PubMed, Web of Science, EMbase, and Cochrane Library, from the inception of the databases up to September 2022. Our data mining was based on keyword searches and combinations, including Oral care (1), Oral health (2), Oral hygiene (3), Oral decontamination (4), Intubation (5), Mechanical ventilation (6), VAP (7), VAP (8), Intensive care unit (9), ICU (10), herb (11), Herbal mouthwash (12), Herbal mouth rinse (13), Natural product (14), Chinese medicine (15), Chinese materia medica (16), Chinese herb (17), RCT (18). The retrieved records were imported into EndNote 21 for screening and deduplication. Any discrepancies between the two investigators were resolved by a third reviewer.

This study employed the PICOS heuristic method to identify eligible studies, which include Population/Participants, Intervention, Comparator, Outcome, and Study design criteria. Eligible participants (P) must be adults receiving mechanical ventilation in the ICU, regardless of gender, region, or any unspecified baseline characteristics. Patients with respiratory infections or other oral diseases were excluded. The eligible intervention (I) in randomized controlled trials involved the use of any natural products for oral care, including raw materials, herbal extracts, finished products, or products containing active ingredients such as traditional Chinese medicine, Miswak, chamomile extract, etc. The eligible comparator (C) in randomized controlled trials included the use of a placebo or standard treatment for oral care, such as CHX, saline, sodium bicarbonate, povidone-iodine, hydrogen peroxide, etc. The eligible outcome (O) in randomized controlled trials was the comparison of VAP incidence (primary outcome) and oral microbial quantity (secondary outcome) among patient groups. In addition to the above criteria, the eligible study design (S) must be a randomized controlled trial. Observational cohort studies, case-control studies, case reports, and reviews were excluded.

### Study quality assessment

We utilized the Grading of Recommendations, Assessment, Development and Evaluations (GRADE) tool [20] to assess the quality of the available evidence. The GRAD-Eprofiler software was employed to assign a rating of high, moderate, low, or very low evidence quality for each included outcome, based on study design, risk of bias, inconsistency, indirectness, imprecision, and publication bias. These factors may increase or decrease the quality of the evidence: (1) risk of bias (downgraded once when less than 75% of analyzed studies are at low risk of bias); (2) inconsistency (downgraded once when I^2^ > 50%); (3) indirectness of evidence (e.g., indirect population, intervention, comparison, or outcome); (4) imprecision with wide confidence intervals; and (5) presence of publication bias also reduces the quality of the evidence.

### Statistical analysis

The meta-analysis adhered to the PRISMA checklist (S2 Table). For binary data, odds ratios (ORs) were used to express treatment effects, while weighted mean differences (WMDs) were used for continuous (mean difference) data. Direct meta-analyses used the 95% confidence interval (CI), while the credible interval (CrI) was used for network meta-analyses. We conducted a network meta-analysis using Bayesian methods [21] in R (version x64 4.2.2) with the gemtc package (version 0.8–2) and rjags package (version 4–6), selecting the model based on comparisons of the DIC, ratio, and I^2^. Inconsistency of results was assessed by the node-splitting method and its Bayesian p-value, comparing the direct and indirect estimates for each comparison. A p-value < 0.05 indicated significant inconsistency. Potential ranking probabilities of treatments were estimated by calculating the rank probability matrix and the SUCRA values, with higher values indicating better efficacy.

## Results

### Selection and characteristics of the studies

**Fig 1:**
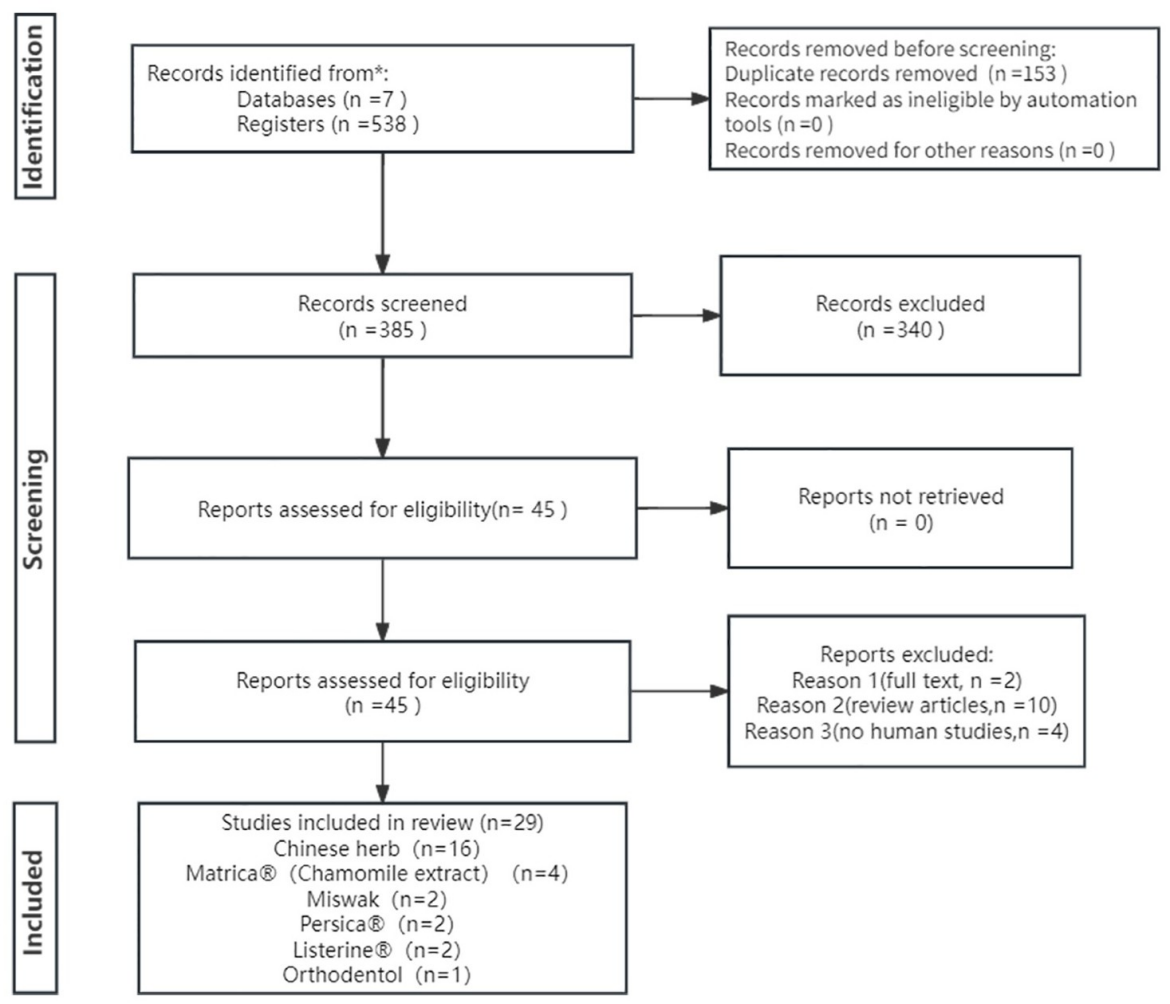
Literature search: Preferred Reporting Items for Systematic Reviews and Meta‑Analyses (PRISMA) consort diagram.

**Fig 2:**
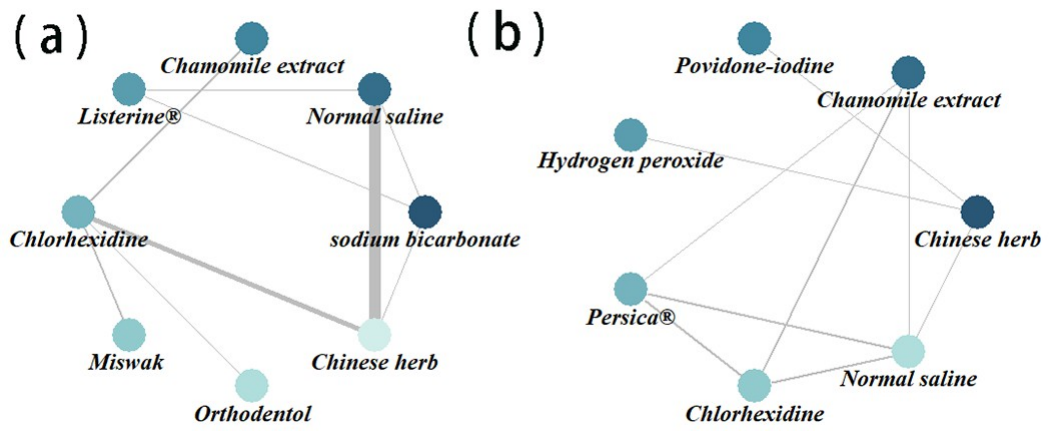
Network geometry graphs for changes on ventilator‑associated pneumonia(a), Colony Number (b).

A total of 538 articles were identified from databases including CNKI, Wanfang, CBM, PubMed, Web of Science, EMbase, and Cochrane Library, with the search period up to September 2023. We used EndNote software to remove duplicates and screened the titles and abstracts to exclude irrelevant studies. Out of 45 articles selected for full-text review, we included 29 studies that met the inclusion and exclusion criteria [22–50]. (Fig 1). Among them, 24 articles reported incidence of VAP [22–45] and six articles reported colony number [40, 46–50]. The included herbal mouthwashes were Miswak (stem of Salvadora persica), Persica^®^ (alcoholic extract of S. persica, Achillea millefolium, and Menthaspicata), Matrica^®^ (Chamomile extract), Listerine^®^ (main components include Menthol, Thymol, and Eucalyptol), Chinese herbs (extracted from multiple herbs), Orthodentol (extract of Khouzestani Savory, containing 30% carvacrol), and other oral care products including CHX, Normal saline, Sodium bicarbonate, Povidone-iodine, and Hydrogen peroxide. Three three-arm studies [22, 29, 48] and one four-arm study [46] were included. (Table 1) (Fig 2).

**Table 1:** Characteristics of studies included.

author, year	age	Design	Treatment description
Berry 2013 [22]	arm1: 58.82(16.7)	Single blind RCT	arm1:NS+Rinsing,12/d+toothbrushing,3/d
	arm2: 54.93(19.5)		arm2:Sodium bicarbonate+Rinsing,12/d+toothbrushing,3/d
	arm3: 59.96(18.0)		arm3:Listerine^®^+Rinsing,2/d+toothbrushing,3/d
Maarefvand 2015 [23]	arm1: 51.63±12.18	RCT	arm1:NS+Children’s toothbrush brushing+ Cleaning with cotton swabs,2/d
	arm2: 45.93±14.11		arm2:Matrica+Brushing with children’s toothbrush+ Cleaning with cotton swabs,2/d
Irani 2019 [24]	arm1: 34.83±13.95	Single blind RCT	arm1:0.2%CHX+cotton swab Wiping,2/d
	arm2: 33.65±13.50		arm2:Miswak+Scrubbing,2/d
Kawyannejad 2020 [25]	arm1: 46.7±14.76	Double blind RCT	arm1:0.2%CHX+Rinsing, 3/d
	arm2: 47.78±11.22		arm2:Orthodentol+Rinsing,3/d
Shen ML 2020 [26]	arm1: 60.1±3.5	RCT	arm1:0.2%CHX+tooth brushing,3/d
	arm2: 59.7±4.6		arm2:Chinese herb+ tooth brushing,3/d
Gao SM 2019 [27]	arm1: 66±5.3	RCT	arm1:0.12%CHX+scrubbing with cotton swabs, 4/d
	arm2: 68±4.9		arm2:Chinese herb+ scrubbing with cotton swabs,4/d
Gao SM 2020 [28]	arm1: 58.92±7.02	RCT	arm1:0.12%CHX+scrubbing with cotton swabs, 4/d
	arm2: 56.45±6.77		arm2:Chinese herb+ scrubbing with cotton swabs, 4/d
Gong CQ 2018 [29]	arm1: 42.52±7.84	RCT	arm1:NS+Wiping, 4/d
	arm2: 46.35±10.28		arm2:NS+Brush teeth with a soft bristled toothbrush, 4/d
	arm3: 46.35±10.28		arm3:Chinese herb+ Rinsing and Brush teeth with a soft brisrled toothbrush, 4/d
Han J 2021 [30]	NR	RCT	arm1:NS+Cleaning with cotton swabs, 4/d
			arm2:Chinese herb+ Cleaning with cotton swabs, 4/d
He YQ 2012 [31]	arm:12–93	RCT	arm1:NS+Cleaning with cotton swabs,4/d
			arm2:Chinese herb+ Rinsingand Cleaning with cotton swabs, 2/d
Jiang JJ 2021 [32]	arm1: 61.29±7.02	RCT	arm1:NS+Cleaning with cotton swabs, 2/d
	arm2: 62.17±8.14		arm2:Chinese herb+ Cleaning with cotton swabs, 2/d
LI QY 2017 [33]	arm1: 76.6±9.8	RCT	arm1:Chinese herb+ Cleaning with cotton swabs, 3/d
	arm2: 76.5±9.6		arm2:NS+Cleaning with cotton swabs,3/d
Liang YD 2020 [34]	arm1: 64.85±10.72	RCT	arm1:NS+tooth brushing,2/d
	arm2: 66.79±9.42		arm2:Chinese herb+ tooth brushing, 2/d
Lu MY 2015 [35]	arm:25–85	RCT	arm1:NS+Rinsing,2/d
			arm2:Chinese herb+ Rinsing, 2/d
Ruan LJ 2013 [36]	arm:36–89	RCT	arm1:NS+Rinsing,2/d
			arm2:Chinese herb+Rinsing,2/d
She YX 2020 [37]	arm:68.9	RCT	arm1:Wiping+0.12%CHX,2/d arm2:Chinese herb+ Wiping, 2/d
Shi CH 2017 [38]	arm1: 57.3±4.2	RCT	arm1:NS+Rinsing,3/d
	arm2: 56.1±3.9		arm2:Chinese herb+Rinsing,3/d
Wang L 2020 [39]	arm1: 59.6±7.2	RCT	arm1:NS+Wiping,2/d
	arm2: 58.6±6.3		arm2:Chinese herb+ tooth brushing+ Rinsing, 2/d
Yang ZX 2014 [40]	arm1: 58.45±5.21	RCT	arm1:2%Hydrogen peroxide+ Cleaning with cotton swabs,4/d
	arm2: 58.01±5.67		arm2:Chinese herb+ Cleaning with cotton swabs, 4/d
Yao YL 2020 [41]	NR	RCT	arm1:NS+Cleaning with cotton swabs,3/d
			arm2:Chinese herb+ Cleaning with cotton swabs, 3/d
Zhang B 2020 [42]	arm1: 75.58±10.57	RCT	arm1:NS+Cleaning and Rinsing,3/d
	arm2: 77.68±9.94		arm2:Chinese herb+ Cleaning and Rinsing, 3/d
Zhang CF 2016 [43]	arm1: 66.7	RCT	arm1:0.12%CHX+Rinsing and Wiping,4/d
	arm2: 66.3		arm2:Chinese herb, Rinsing and Wiping, 4/d
Rezvani 2018 [44]	arm1: 59.18±20.15	Double blind RCT	arm1:0.2%CHX+Rinsing and scrubbing with gauze,2/d
	arm2: 60.78±18.41		arm2:Matrika+Rinsing and scrubbing with gauze,2/d
Hafez 2015 [45]	arm1: 38.10±19.759	RCT	arm1:0.12%CHX+tooth brushing,6/d
	arm2: 45.65±19.381		arm2:Miswak+tooth brushing,6/d
Haidari 2013 [46]	arm1: 49,6±1,31	Double blind RCT	arm1:0.2%CHX+Rinsing, 4/d
	arm2: 52,35±1,51		arm2:0.12%CHX+tooth brushing,6/d
	arm3: 50,45±1,13		arm3:10%Matrica^®^+Rinsing, 4/d
	arm4: 52,7±1,24		arm4:NS+Rinsing, 4/d
Baradari 2012 [47]	arm1: 47.52±7.1	Double blind RCT	arm1:0.2%CHX+Rinsing, 4/d
	arm2: 47.56±8.6		arm2:Matrica^®^ (Chamomile extract) +Rinsing, 4/d
Taraghi 2011 [48]	arm1: 49.6±1.31	Double blind RCT	arm1:0.2%CHX+Rinsing, 4/d
	arm2: 52.35±1.51		arm2:NS+Wiping, 4/d
	arm3: 52.7±1.24		arm3:10%persica^®^Wiping, 4/d
XU DQ 2021 [49]	arm1: 58.01±5.67	RCT	arm1:0.1%Povidone-iodine+Rinsing and Brushing with children’s toothbrush, 3/d
	arm2: 58.45±5.21		arm2:Chinese herb+ Rinsing and Brushing with children’s toothbrush, 3/d
Zhang HY 2014 [50]	arm1: 70±8.68	RCT	arm1:NS+Rinsing and Wiping, 3/d
	arm2: 70±8.99		arm2:Chinese herb+ Rinsing and Wiping, 3/d

**Note:** NS means Normal saline; CHX means chlorhexidine; RCT Randomized Controlled Trial.

### Incidence of VAP

The direct and indirect comparison results showed consistency. Chinese herb was superior to Orthodentol (OR: 0.11, 95% CI: 0.02–0.51), CHX (OR: 0.32, 95% CI: 0.17–0.59), Listerine^®^ (OR: 0.16, 95% CI: 0.05–0.59), Chamomile extract (OR: 0.33, 95% CI: 0.13–0.79), Normal saline (OR: 0.16, 95% CI: 0.1–0.24), and Sodium bicarbonate (OR: 0.2, 95% CI: 0.07–0.52). Miswak was superior to Orthodentol (OR: 0.11, 95% CI: 0.02–0.64), Sodium bicarbonate (OR: 0.19, 95% CI: 0.14–0.93), Normal saline (OR: 0.16, 95% CI: 0.14–0.53), Listerine^®^ (OR: 0.17, 95% CI: 0.03–0.97), and CHX (OR: 0.33, 95% CI: 0.1–0.92). The other comparisons were not significant. (Table 2).

**Table 2:** Indirect comparison of oral care solutions.

**A)**								
**VAP rate**	**Sodium bicarbonate**	**Normal saline**	**Chamomile extract**	**Listerine** ^**®**^	**Chlorhexidine**	**Miswak**	**Orthodentol**	**Chinese herb**
**Sodium bicarbonate**	**-**	1.18(0.41,3.2)	0.43(0.09,1.99)	1.17(0.32,4.07)	0.5(0.15,1.75)	**0.13(0.02,0.75)**	1.51(0.23,10.99)	**0.2(0.07,0.54)**
**Normal saline**	0.85(0.31,2.41)	**-**	0.37(0.11,1.15)	0.99(0.27,3.42)	**0.43(0.2,0.92)**	**0.11(0.02,0.48)**	1.27(0.25,7.05)	**0.17(0.11,0.25)**
**Chamomile extract**	2.31(0.5,10.66)	2.71(0.87,8.8)	**-**	2.68(0.5,14.67)	1.15(0.5,2.79)	0.31(0.06,1.36)	3.47(0.65,19.68)	0.45(0.15,1.35)
**Listerine** ^**®**^	0.85(0.25,3.15)	1.01(0.29,3.65)	0.37(0.07,1.99)	**-**	0.43(0.1,1.92)	**0.11(0.01,0.78)**	1.29(0.17,10.84)	**0.17(0.05,0.63)**
**Chlorhexidine**	2(0.57,6.79)	**2.34(1.09,5.01)**	0.87(0.36,2.01)	2.34(0.52,9.97)	**-**	**0.27(0.07,0.91)**	2.98(0.72,13.95)	**0.39(0.2,0.75)**
**Miswak**	**7.55(1.33,47.46)**	**8.92(2.06,42.9)**	3.25(0.74,16.56)	**8.75(1.29,66.76)**	**3.75(1.1,14.97)**	**-**	**11.41(1.75,87.4)**	1.49(0.36,6.87)
**Orthodentol**	0.66(0.09,4.39)	0.79(0.14,4.04)	0.29(0.05,1.55)	0.78(0.09,5.92)	0.34(0.07,1.38)	**0.09(0.01,0.57)**	**-**	**0.13(0.02,0.63)**
**Chinese herb**	**5.08(1.84,14.38)**	**5.97(4.07,9.05)**	2.2(0.74,6.52)	**5.97(1.6,21.2)**	**2.55(1.34,4.98)**	0.67(0.15,2.76)	**7.61(1.59,40.96)**	**-**
**B)**								
**Colony Number**	**Chinese herb**	**Chamomile extract**	**Povidone-iodine**	**Hydrogen peroxide**	**Persica** ^**®**^	**Chlorhexidine**	**Normal saline**	
**Chinese herb**	**-**	1.64(0.82,3.33)	**3.32(2.09,5.26)**	1.04(0.56,1.91)	1.63(0.82,3.2)	1.04(0.53,2.03)	**1.91(1.04,3.5)**	
**Chamomile extract**	0.61(0.3,1.22)	**-**	2.02(0.87,4.62)	0.63(0.25,1.58)	0.99(0.68,1.41)	**0.63(0.46,0.85)**	1.17(0.8,1.67)	
**Povidone-iodine**	**0.3(0.19,0.48)**	0.49(0.22,1.15)	-	**0.31(0.15,0.66)**	0.49(0.22,1.1)	**0.31(0.14,0.7)**	0.58(0.27,1.22)	
**Hydrogen peroxide**	0.96(0.52,1.77)	1.58(0.63,4.01)	3.19(1.51,6.82)	**-**	1.56(0.63,3.87)	1(0.4,2.47)	1.84(0.78,4.33)	
**Persica** ^**®**^	0.62(0.31,1.22)	1.01(0.71,1.46)	2.04(0.91,4.64)	0.64(0.26,1.58)	**-**	**0.64(0.47,0.87)**	1.18(0.85,1.62)	
**Chlorhexidine**	0.97(0.49,1.9)	**1.58(1.18,2.16)**	**3.21(1.42,7.2)**	1(0.41,2.49)	**1.57(1.16,2.12)**	-	**1.85(1.35,2.52)**	
**Normal saline**	**0.52(0.29,0.96)**	0.85(0.6,1.25)	1.74(0.82,3.69)	0.54(0.23,1.28)	0.85(0.62,1.17)	**0.54(0.4,0.74)**	**-**	

**Note: A)** Random-effects model. Negative values indicate improvement in clinical response. Changes that are statistically significant are shown in bold. **B)** The upper right triangle provides the pooled risk ratios from pairwise comparisons (column interventions relative to row interventions), while the lower left triangle summarizes the standardized mean differences and raw mean differences from network meta-analysis (row interventions relative to column interventions). Bold values indicate statistical significance with p<0.05.

### Colony number

The direct and indirect comparison results showed consistency. In terms of reducing colony count, Chinese herb (OR: 0.3, 95% CI: 0.19–0.48) and CHX (OR: 0.54, 95% CI: 0.4–0.74) were superior to Normal saline. Hydrogen peroxide was superior to Povidone-iodine (OR: 0.31, 95% CI: 0.15–0.66). Chinese herb was superior to Povidone-iodine (OR: 0.33, 95% CI: 0.19–0.48). CHX was superior to Chamomile extract (OR: 0.63, 95% CI: 0.46–0.85), Povidone-iodine (OR: 0.31, 95% CI: 0.14–0.7), and Persica^®^ (OR: 0.64, 95% CI: 0.47–0.87). The remaining comparisons were not significant. (Table 2).

### Treatment ranking

**Fig 3:**
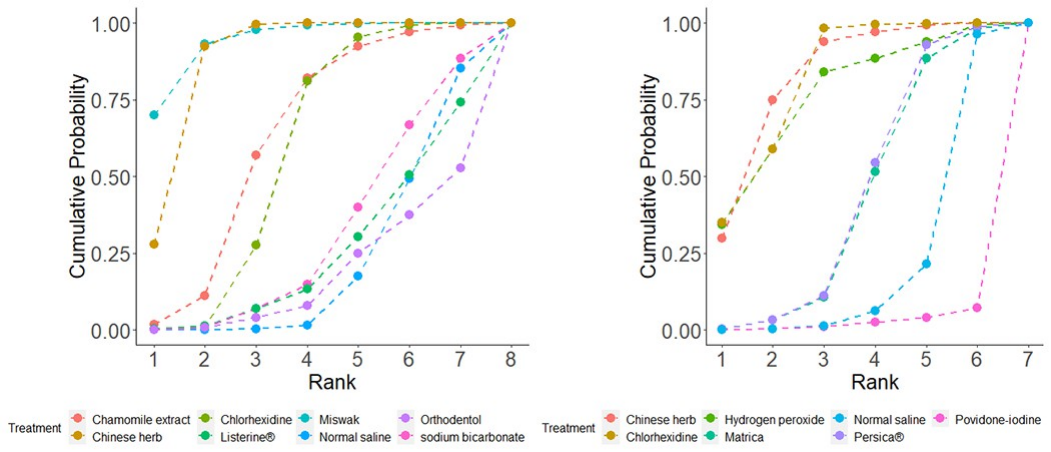
Treatment ranking for each assessed outcome: Incidence of ventilator‑associated pneumonia(a), Colony Number (b).

Based on the research results, rank probabilities were used to generate a ranking graph (Fig 3). The rank. Probability function was used to determine the top two interventions for each outcome measure and compared with the SUCRA (Rank) results. The ranking of interventions in reducing VAP incidence were as follows: Miswak (94.25%), Chinese herb(88.25%), Chamomile extract (62.94%), CHX (57.72%), Sodium bicarbonate (31.14%), Listerine^®^ (25.2%), Normal saline (21.97%), Orthodentol (18.23%). The ranking of interventions in reducing Colony Number were as follows: Chinese herb (82.48%), CHX (81.93%), Hydrogen peroxide (76.56%), Persica^®^ (43.48%), Chamomile extract (42.07%), Normal saline (20.94%), Povidone-iodine (2.54%).

### Publication bias

After evaluating the funnel plot and Egger’s test (VAP rate: z = -2.3167, p = 0.0205; Colony Number: z = -0.5110, p = 0.6094), the results showed significant asymmetry in the funnel plot. Although the test result showed no bias in VAP rate, we used the trim-and-fill method and found that 6 studies with non-significant results were missing, indicating some degree of publication bias (S1 Fig).

### Recommendation of evidence

According to the GRADE evaluation guide [20], grade-profiler was used to evaluate literature quality. All levels of evidence supporting this result were rated moderate to very low. Due to the limitations of the study, the included herb related studies were all from China and Iran, and there was no double-blind study on Chinese herb related studies. Therefore, the results presented in this NMA should be treated with caution. (S1 Table).

## Discussion

**Fig 4:**
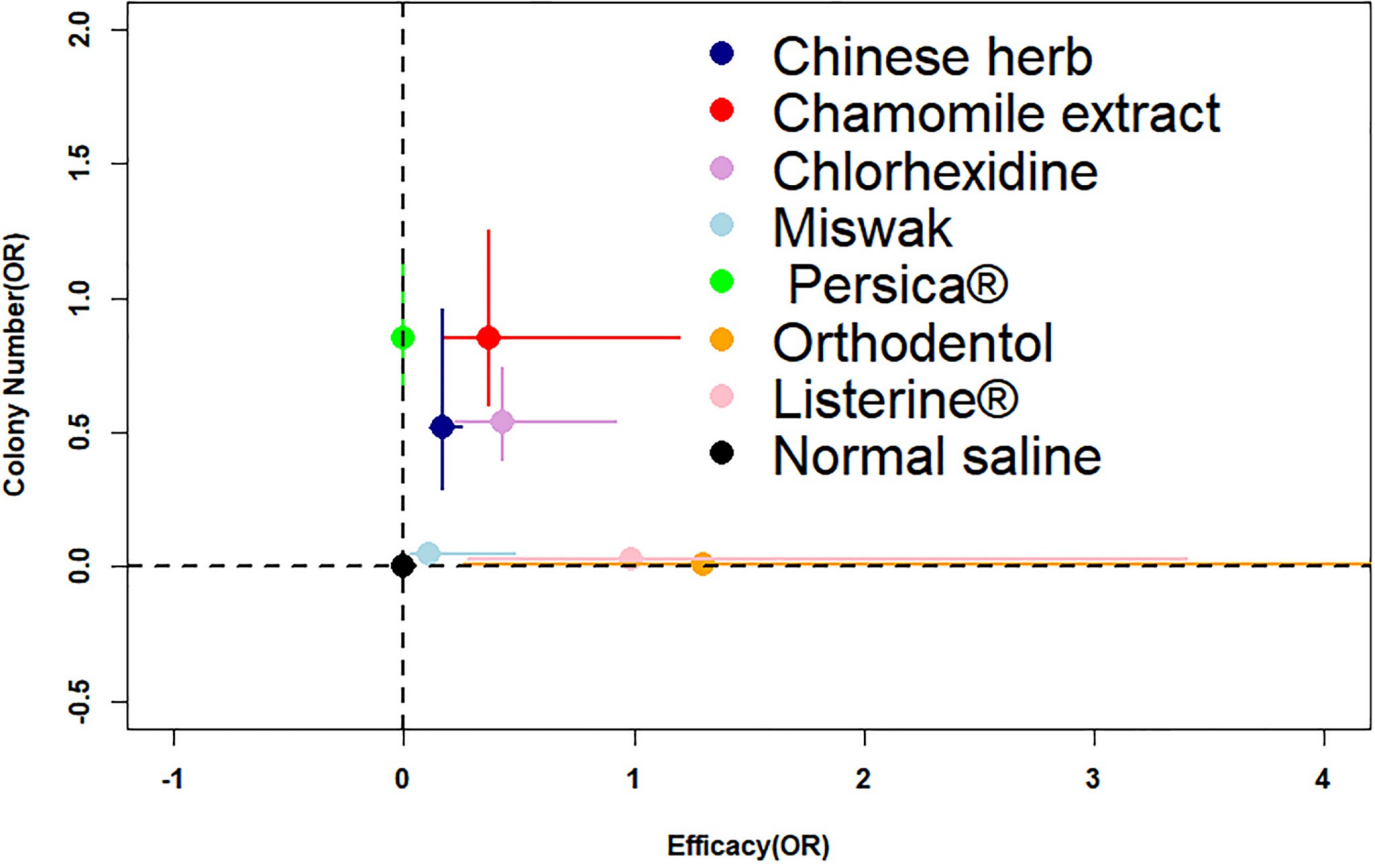
A two-dimensional graph showing the incidence rate (OR) and Colony Number (OR) of Herbal oral care products.

After screening, we included the following herbal mouthwashes: Miswak (stem of Salvadora persica), Persica^®^ (alcoholic extract of S. persica, Achillea millefolium, and Mentha spicata), Matrica^®^ (Chamomile extract), Listerine^®^ (main components include Menthol, Thymol, and Eucalyptol). Fig 4 shows the two-dimensional plot of efficacy and colony count of all herbal and natural oral care products.

Consistent with the results of clinical double-blind trials [44], we found that Matrica^®^ significantly reduced microbial counts but did not affect the incidence of VAP. In contrast, traditional Chinese medicine and Miswak were more effective than CHX in reducing the incidence of VAP, according to our network meta-analysis comparison. Therefore, we consider Miswak and Matrica^®^ as the most promising herbal oral care products to potentially replace CHX. However, the safety and feasibility of traditional Chinese medicine require further high-quality research for confirmation. It is important to note that our study provides directions for future research rather than definitive conclusions.

Initially, it is important to clarify that although Chinese herb showed superiority in our NMA, the quality of research on Chinese herb is extremely low, with most studies lacking blinding and randomization. Chinese herb is a complex multi-herb preparation, containing several herbs such as honeysuckle [51] and peppermint [52], and we speculate that the efficacy of Chinese herb may come from these herbs. However, Chinese herb requires boiling and residue removal from multiple herbs, making it difficult to ensure quality and may lead to differences in efficacy and potential safety risks. Furthermore, it is not feasible for large-scale promotion since the included studies mostly used self-made Chinese herb preparations. Therefore, we do not recommend the use of Chinese herbs. All studies have not mentioned any potential side effects and there is no related research on the matter. We believe that the safety of Chinese herbs is questionable.

According to the node-splitting method, there was no significant inconsistency among the included studies, and the overall network structure was reliable (P>0.05). (S2 and S3 Figs).

Our results are similar to some of the findings in a recent systematic review [53]. However, our study showed that Chamomile extract had a significant advantage over CHX in reducing Colony Number (OR: 0.63, 95% CI: 0.46–0.85), but no significant advantage over CHX in reducing the incidence of VAP (OR: 1.15, 95% CI: 0.5–2.79), which is different from the clinical trial results [23]. We believe that the reason for this contradictory conclusion may be due to limitations in our study’s inclusion criteria, as we did not conduct a detailed meta-analysis of Colony Number for VAP pathogens such as Staphylococcus aureus and Pseudomonas aeruginosa [54]. We only compared the total number of colonies, which may explain the difference in our results compared to some clinical trials. In addition, the number of articles included in our study was limited, with most coming from China and Iran.

Apart from multi-ingredient mouthwashes, we believe that Miswak is an oral care product that can replace CHX. Although there are currently no long-term studies or large-scale prospective studies on the use of Miswak, Miswak has a long history of use as a traditional oral care product [55, 56]. In addition, the World Health Organization recommends and encourages the use of Miswak as an effective oral hygiene practice. Most importantly, Miswak can simultaneously act as a toothbrush and oral care solution, and its effectiveness in improving oral health in the general population has been demonstrated [57].

Despite being less effective than CHX in both NMA and clinical trials, Matrica^®^ has shown to be more effective than saline in reducing Colony Number and VAP incidence in ICU patients, consistent with clinical results [47]. Considering the antimicrobial properties of Matrica^®^ herbal mouthwash demonstrated in current and previous studies, it has been shown to have a beneficial effect in reducing plaque and gingivitis [58], with stronger resistance and significantly fewer side effects compared to chemical mouthwashes. Chamomile extract is considered safe for oral care, with the only potential short-term severe side effect being allergic reactions [59]. Further research is needed to investigate long-term side effects and mortality rates. Chamomile extract may have the potential to prevent VAP in ICU patients.

As for the three herbal oral care products Listerine^®^, Orthodentol, and Persica^®^, our results are similar to clinical studies [22, 25, 47]. Although they are also superior to Normal saline in terms of antibacterial ability, there are currently significant issues with these products, and we do not recommend them. Firstly, the safety of Listerine^®^ is very questionable as it contains alcohol, and its component thymol has cytotoxicity. Oral microorganisms can metabolize ethanol into acetaldehyde, causing DNA damage, and may even be associated with oral cancer, raising concerns about the dosage and concentration used [60]. Secondly, Orthodentol is an herbal mouthwash with a natural formula, and its component Khouzestani Savory is a native plant in Iran that contains 30% carvacrol. Although studies have shown that it has many antibacterial effects and significant effects on toothache [61], there are no short-term and long-term in vivo toxicology data available for carvacrol. In vitro experiments have provided sufficient evidence of mild to moderate toxicity on the cellular level [62]. Additionally, Persica^®^ herbal mouthwash contains three medicinal plants, Persica Salvadora, Milenrama, and mint. These plants can be consumed without short-term safety concerns, which is an advantage. However, some studies have shown that it does not seem to have an effect on gram-negative bacteria, and our NMA results also show that it is not ideal [63].

Despite numerous studies demonstrating the side effects of CHX and guidelines recommending against its use, it is still widely used in various patient populations, especially ICU patients [64, 65]. Currently, although there are many herbal oral care products available, there is still limited research on herbal oral care products for ICU mechanically ventilated patients, particularly with regards to long-term follow-up studies on side effects and mortality rates. Therefore, we believe that further research is needed to explore Miswak, chamomile extract, and Chinese herbs in greater depth. While the safety of Persica^®^ can be ensured, its efficacy appears to be lower. Further research is needed to confirm the safety of using Listerine^®^ and Orthodentol in MV patients.

## Limitation

This study investigates the efficacy of herbal oral care products on ICU patients. However, several limitations need consideration. Firstly, the data for this study is derived from published literature, and potential publication bias was assessed through funnel plot and Egger’s test. Secondly, the broad inclusion criteria resulted in varying study qualities, with only six studies employing blinding methods. Some studies lacked clarity on randomization methods, potentially impacting the reliability of results. Thirdly, variations in the frequency of herbal oral care product usage and accompanying oral care protocols introduce heterogeneity, potentially affecting their effectiveness in preventing ventilator-associated pneumonia and reducing oral microbial counts. Lastly, the study exclusively evaluates the impact of herbal oral care products on the incidence of ventilator-associated pneumonia and oral microbial counts. Important outcomes related to oral care, such as long-term safety, oral mucosal damage, gingivitis, were not addressed. These outcomes may influence the prognosis and comfort of mechanically ventilated patients, necessitating further clinical research to fill these data gaps.

Therefore, the conclusions drawn from this study should be interpreted cautiously. Future research demands high-quality, large-sample, multicenter, double-blind randomized controlled trials to assess the long-term effects and safety of herbal oral care products. Additionally, investigating other relevant outcomes is crucial for providing robust evidence supporting the application of herbal oral care products in mechanically ventilated patients.

## Conclusion

Based on our network meta-analysis, we have observed that Chinese herbal medicine and Miswak are superior to CHX in reducing the incidence of VAP. However, the safety and feasibility of traditional Chinese herbal medicine require further high-quality research for validation. Simultaneously, Matrica^®^ demonstrates a significant reduction in microbial counts but does not exhibit a significant advantage in lowering the incidence of VAP. This observation aligns with the results of clinical double-blind trials. Therefore, we identify Miswak and Matrica^®^ as promising herbal oral care products with the potential to replace CHX. It is essential to emphasize that our study provides guidance for future research rather than conclusive determinations.

## Supporting information

S1 Fig Funnel plot of the network meta-analysis indicating publication bias. (TIF)

S2 Fig Node-splitting P value. (TIF)

S3 Fig Inconsistency test result. (TIF)

S1 Table Quality assessment by GRADE. (DOCX)

S2 Table PRISMA 2020 checklist. (DOC)
