# Supplementary figures and images for: Herbal oral care products for the prevention of ventilator-associated pneumonia: A systematic review and network meta-analysis of randomised trials
Source: PLoS One. 2024 Jun 7;19(6):e0304583. doi: 10.1371/journal.pone.0304583 (PMC11161024; doi:10.1371/journal.pone.0304583)

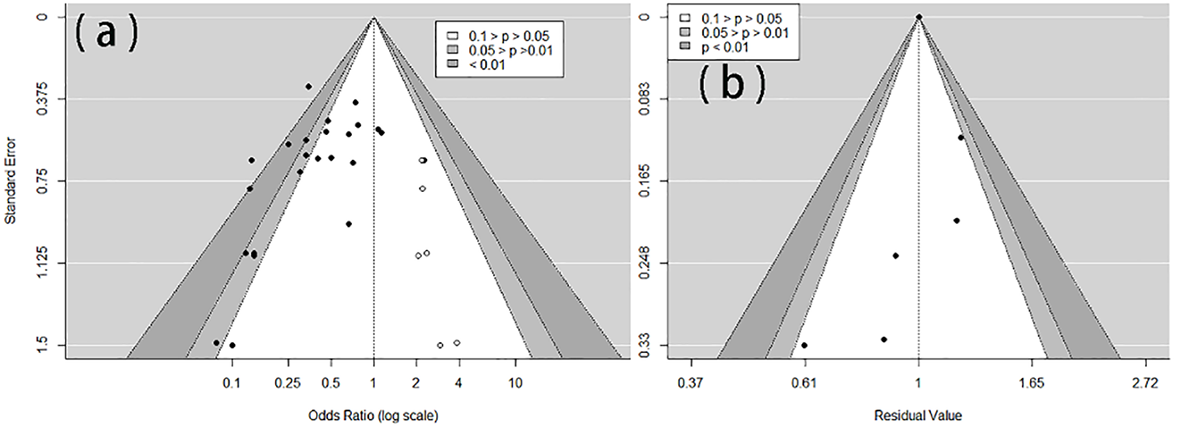

Supplement: S1 Fig — (TIF) [file pone.0304583.s001.tif]

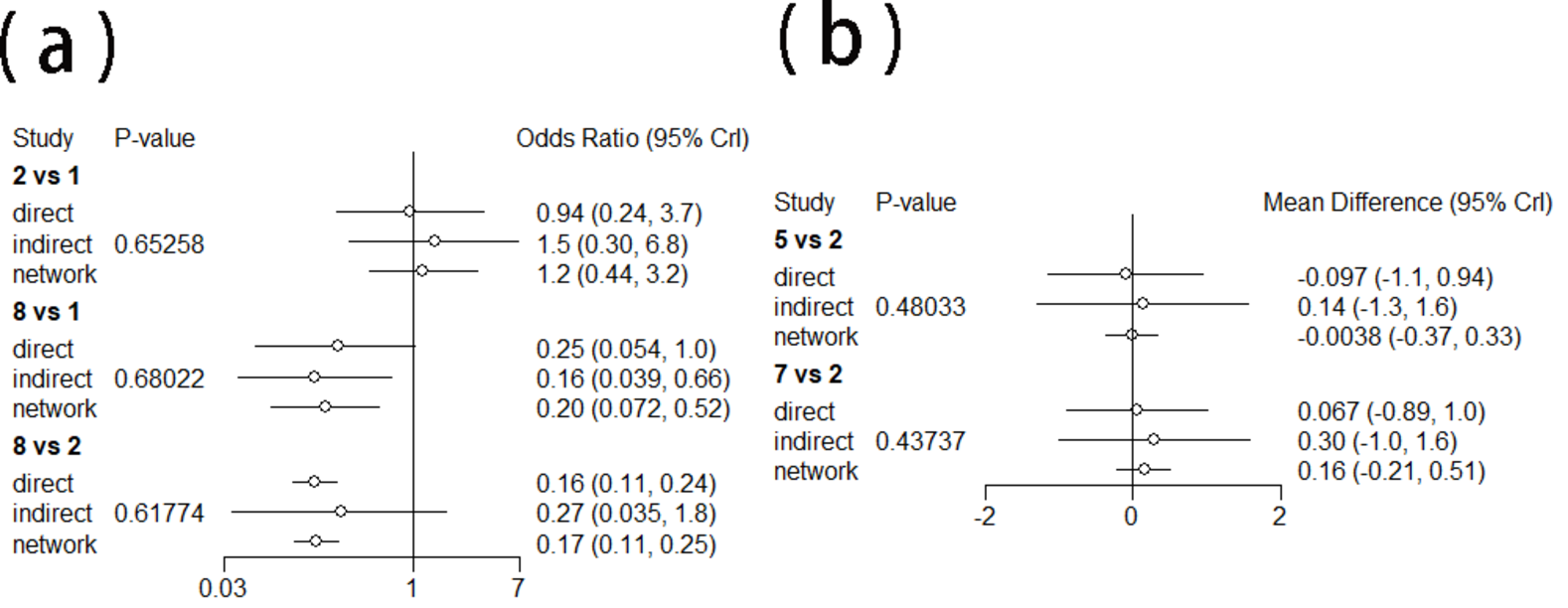

Supplement: S2 Fig — (TIF) [file pone.0304583.s002.tif]

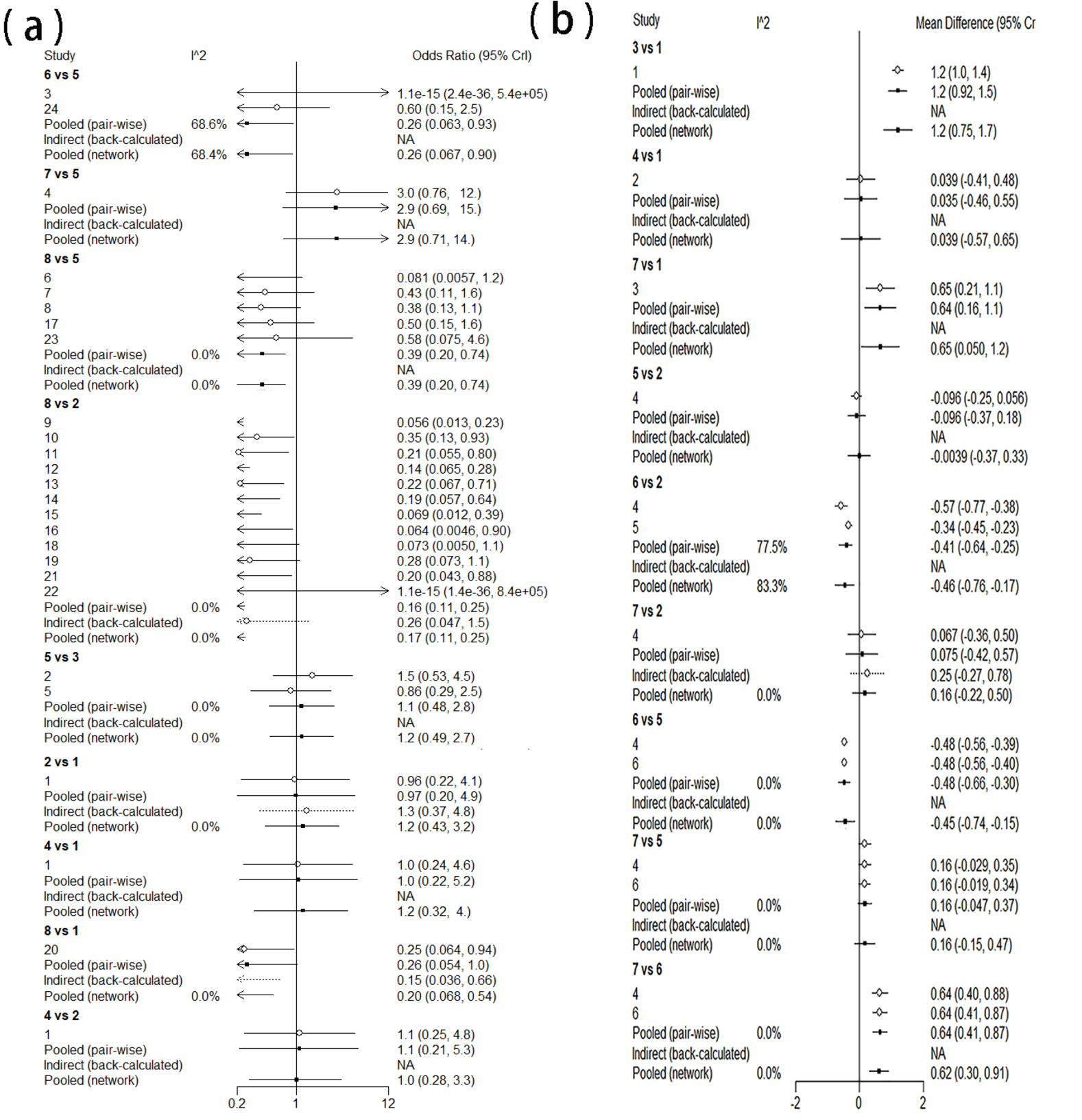

Supplement: S3 Fig — (TIF) [file pone.0304583.s003.tif]
