# Supplementary material for: Herbal oral care products for the prevention of ventilator-associated pneumonia: A systematic review and network meta-analysis of randomised trials
Source: PLoS One. 2024 Jun 7;19(6):e0304583. doi: 10.1371/journal.pone.0304583 (PMC11161024; doi:10.1371/journal.pone.0304583)
Supplement: S1 Table — (DOCX) [file pone.0304583.s004.docx]

**ADDITIONAL FILE 4. Quality assessment by GRADE.**

| **Certainty assessment** | | | | | | | **NO of patients** | | **Effect** | | **Certainty** | **Importance** |
| --- | --- | --- | --- | --- | --- | --- | --- | --- | --- | --- | --- | --- |
| **NO of studies** | **Study design** | **Risk of bias** | **Inconsistency** | **Indirectness** | **Imprecision** | **Other considerations** | **VAP related indicators** | **[health problem]** | **Relative (95% CI)** | **Absolute (95% CI)** |  |  |
| **Incidence of VAP** | | | | | | | | | | | | |
| 24 | randomised trials | very serious^a^ | serious^b^ | serious^c^ | not serious | none | 319/2787 (11.4%) | 697/2427 (28.7%) | **OR 0.30** (0.26 to 0.35) | **179 fewer per 1,000** (from 192 fewer to 164 fewer) | ⨁◯◯◯ Very low | important |
|  |  |  |  |  |  |  |  | 33.3% |  | **203 fewer per 1,000** (from 218 fewer to 184 fewer) |  |  |
| **Incidence of VAP - Miswak** | | | | | | | | | | | | |
| 2 | randomised trials | not serious | not serious | not serious | serious^d^ | none | 5/55 (9.1%) | 13/55 (23.6%) | **OR 0.31** (0.10 to 0.98) | **149 fewer per 1,000** (from 206 fewer to 4 fewer) | ⨁⨁⨁◯ Moderate | important |
|  |  |  |  |  |  |  |  | 26.1% |  | **162 fewer per 1,000** (from 227 fewer to 4 fewer) |  |  |
| **Incidence of VAP - Chinese herb** | | | | | | | | | | | | |
| 18 | randomised trials | very serious^a^ | not serious | not serious | serious^d^ | none | 112/847 (13.2%) | 298/850 (35.1%) | **OR 0.22** (0.17 to 0.29) | **244 fewer per 1,000** (from 267 fewer to 215 fewer) | ⨁◯◯◯ Very low | important |
|  |  |  |  |  |  |  |  | 34.2% |  | **239 fewer per 1,000** (from 261 fewer to 211 fewer) |  |  |
| **Incidence of VAP - Chlorhexidine** | | | | | | | | | | | | |
| 10 | randomised trials | very serious^a^ | not serious | not serious | serious^d^ | none | 53/429 (12.4%) | 82/429 (19.1%) | **OR 0.57** (0.39 to 0.84) | **72 fewer per 1,000** (from 107 fewer to 26 fewer) | ⨁◯◯◯ Very low | important |
|  |  |  |  |  |  |  |  | 18.6% |  | **71 fewer per 1,000** (from 104 fewer to 25 fewer) |  |  |
| **Incidence of VAP - Chamomile extract** | | | | | | | | | | | | |
| 2 | randomised trials | not serious | not serious | not serious | very serious^e^ | none | 18/75 (24.0%) | 22/75 (29.3%) | **OR 0.75** (0.36 to 1.58) | **56 fewer per 1,000** (from 163 fewer to 103 more) | ⨁⨁◯◯ Low | important |
|  |  |  |  |  |  |  |  | 31.7% |  | **59 fewer per 1,000** (from 174 fewer to 106 more) |  |  |
| **Incidence of VAP - Sodium bicarbonate** | | | | | | | | | | | | |
| 2 | randomised trials | serious^f^ | serious^b^ | not serious | serious^e^ | none | 32/291 (11.0%) | 33/169 (19.5%) | **OR 0.62** (0.29 to 1.30) | **64 fewer per 1,000** (from 130 fewer to 45 more) | ⨁◯◯◯ Very low | important |
|  |  |  |  |  |  |  |  | 45.7% |  | **114 fewer per 1,000** (from 261 fewer to 65 more) |  |  |
| **Incidence of VAP - Listerine®** | | | | | | | | | | | | |
| 1 | randomised trials | not serious | not serious | not serious | serious^e^ | none | 12/260 (4.6%) | 6/138 (4.3%) | **OR 1.06** (0.39 to 2.90) | **2 more per 1,000** (from 26 fewer to 73 more) | ⨁⨁⨁◯ Moderate | important |
|  |  |  |  |  |  |  |  | 4.4% |  | **3 more per 1,000** (from 26 fewer to 74 more) |  |  |
| **Incidence of VAP - Normal saline** | | | | | | | | | | | | |
| 13 | randomised trials | very serious^a^ | not serious | not serious | serious^d^ | none | 78/800 (9.8%) | 239/681 (35.1%) | **OR 0.19** (0.14 to 0.25) | **258 fewer per 1,000** (from 281 fewer to 232 fewer) | ⨁◯◯◯ Very low | important |
|  |  |  |  |  |  |  |  | 35.0% |  | **257 fewer per 1,000** (from 280 fewer to 231 fewer) |  |  |
| **Incidence of VAP - Orthodentol** | | | | | | | | | | | | |
| 1 | randomised trials | not serious | not serious | not serious | serious^d^ | none | 9/30 (30.0%) | 4/30 (13.3%) | **OR 2.79** (0.75 to 10.33) | **167 more per 1,000** (from 30 fewer to 480 more) | ⨁⨁⨁◯ Moderate | important |
|  |  |  |  |  |  |  |  | 13.3% |  | **167 more per 1,000** (from 30 fewer to 480 more) |  |  |
| **Colony Number** | | | | | | | | | | | | |
| 6 | randomised trials | very serious^a^ | not serious | serious^c^ | very serious^e^ | none | 492 | 572 | - | MD **0.09 lower** (0.33 lower to 0.15 higher) | ⨁◯◯◯ Very low | important |
| **Colony Number - Chinese herb** | | | | | | | | | | | | |
| 3 | randomised trials | very serious^a^ | not serious | serious^b^ | serious^e^ | none | 96 | 96 | - | MD **0.65 lower** (1.37 lower to 0.06 higher) | ⨁◯◯◯ Very low | important |
| **Colony Number - Chlorhexidine** | | | | | | | | | | | | |
| 3 | randomised trials | serious^f^ | not serious | not serious | not serious | none | 90 | 110 | - | MD **0.24 higher** (0.13 higher to 0.36 higher) | ⨁⨁⨁◯ Moderate | important |
| **Colony Number - Hydrogen peroxide** | | | | | | | | | | | | |
| 1 | randomised trials | serious^f^ | not serious | not serious | serious^d^ | none | 31 | 31 | - | MD **0.04 lower** (0.49 lower to 0.41 higher) | ⨁⨁◯◯ Low | important |
| **Colony Number - Persica®** | | | | | | | | | | | | |
| 2 | randomised trials | not serious | not serious | not serious | serious^d^ | none | 60 | 80 | - | MD **0.18 higher** (0.08 higher to 0.28 higher) | ⨁⨁⨁◯ Moderate | important |
| **Colony Number - Chamomile extract** | | | | | | | | | | | | |
| 3 | randomised trials | not serious | not serious | not serious | serious^d^ | none | 90 | 110 | - | MD **0.24 higher** (0.13 higher to 0.36 higher) | ⨁⨁⨁◯ Moderate | important |
| **Colony Number - Normal saline** | | | | | | | | | | | | |
| 3 | randomised trials | serious^f^ | not serious | serious^b^ | very serious^e^ | none | 75 | 95 | - | MD **0.01 higher** (0.28 lower to 0.29 higher) | ⨁◯◯◯ Very low | important |
| **Colony Number - Povidone-iodine** | | | | | | | | | | | | |
| 1 | randomised trials | serious^f^ | not serious | not serious | not serious | none | 50 | 50 | - | MD **1.2 lower** (1.36 lower to 1.04 lower) | ⨁⨁⨁◯ Moderate | important |

**CI:** confidence interval; **MD:** mean difference; **OR:** odds ratio

#### Explanations

a. Three to more studies included had high risk of bias.

b. I2>50 with moderate heterogeneity

c. If more than 50% of studies included had moderate or high indirectness.

d. If more than 50% of studies included presented wide confidence intervals or not statistically significant effects

e. If more than 50% of studies included presented wide confidence intervals and not statistically significant effects.

f. At least one study included had high risk of bias.
